# Adolescents’ Emotion Regulation Strategies Questionnaire–Extended: Further Development and Associations With Mental Health Problems in Adolescence

**DOI:** 10.1177/10731911231164619

**Published:** 2023-04-13

**Authors:** Gustaf Rådman, Benjamin Claréus, Daiva Daukantaitė

**Affiliations:** 1 Lund University, Sweden

**Keywords:** emotion regulation, adolescence, psychopathology, psychometrics, questionnaire

## Abstract

Emotion regulation (ER) is implicated in a range of psychopathologies and behavioral problems that are prevalent or have their initial onset in adolescence. In this study, we aim to evaluate the psychometric properties (factor structure, internal consistency, and construct validity) of the *Adolescents’ Emotion Regulation Strategies Questionnaire–Extended* (AERSQ-E), a modified and extended version of an ER instrument developed by Zhou et al. Across six sub-studies using data from different Swedish adolescent community samples (1,104 students in total), we generated and validated a 23-item version containing six subscales: rumination/negative thinking, positive reorientation, creative expression, aggressive outlet, social support, and distraction. Assessing test–retest reliability, internal consistency, measurement invariance as well as convergent and discriminant validity, we could establish, with some limitations, the general reliability and validity of the AERSQ-E as a valid measure of ER strategies for use in adolescence.

Emotions are intimately bound to regulatory processes that continuously monitor and adjust their expression (Yih et al., 2018). Emotion regulation (ER) describes these extrinsic and intrinsic processes that monitor the demands of the current environment, evaluate what constitutes a contextually appropriate response, and modify emotional reactions accordingly (Thompson, 1994). Hence, ER is crucially important for adaptive functioning in that it helps modulate the intensity and temporality of an emotional response in proportion to current events and in accordance with personal goals (Aldao, 2013).

ER theoretically implies both the regulation of negative and positive affect. However, dysfunctions in the regulation of negative affect are particularly linked to psychopathology (Beauchaine, 2015; Cole et al., 2017; McLaughlin et al., 2011) and have been suggested as a transdiagnostic factor in the development and maintenance of both internalizing and externalizing psychological problems (Aldao et al., 2016). Difficulties in regulating negative affect have been implicated in a wide range of specific psychopathologies and destructive behaviors (Aldao et al., 2010). Some examples include non-suicidal self-injury (NSSI) (Andover & Morris, 2014; Wolff et al., 2019); eating disorders and disordered eating (DE) (Brockmeyer et al., 2014; Dingemans et al., 2017); mood and anxiety disorders (Hofmann et al., 2012); and aggression (Roberton et al., 2012; Sullivan et al., 2010).

Many of these psychological and behavioral problems have their initial onset in adolescence, a period of rapid change in, not least, the underlying structures supporting ER (Ahmed et al., 2015). Thus, identifying functional and dysfunctional patterns of ER in this critical period is of high clinical relevance, as is by consequence the need for valid and reliable instruments to measure ER, particularly in adolescence. Although numerous self-report instruments have been developed to assess ER (e.g., Garnefski et al., 2001; Gratz & Roemer, 2004; Grob & Smolenski, 2005; Gross & John, 2003; Hofmann et al., 2016; Hofmann & Kashdan, 2010; Phillips & Power, 2007; Zeman et al., 2001; Zhou et al., 2020), the majority of these have been developed for measuring ER in adults or children, with only a few specifically targeting an adolescent demographic.

In this study, we sought to evaluate the psychometric properties of a further developed instrument, the *Adolescents’ Emotion Regulation Strategies Questionnaire* (AERSQ), a scale tailored to measure ER strategies used to regulate negative affect in adolescents (Zhou et al., 2020). We sought to improve its psychometric properties and to extend it by adding and validating an additional ER strategy measure—aggressive outlet—which may be especially important for adolescents who self-injure (Daukantaitė et al., 2019; Tang et al., 2013). As part of the validating procedure for this extended version (referred to as AERSQ-E), we also explore the associations between adolescents’ ER strategies and problematic behaviors such as NSSI and DE, as well as mental health problems across several community samples of Swedish adolescents.

## Classification of Emotion Regulation Strategies

There are many mechanisms by which we regulate our emotions and several dimensions by which we conceptually classify ER strategies. The relationship between ER strategies and psychopathology varies depending on the specific strategy (Aldao et al., 2010). Thus, a key target of ER research is the mapping of ER profiles and how they relate to positive and negative indices of mental health. This, in turn, requires tools that assess a broad range of ER types.

One way to differentiate between different ER strategies is based on the processes they primarily engage in. For instance, some ER strategies are more oriented toward changes in cognition (e.g., cognitive reappraisal, rumination) and some more oriented toward behavioral changes (e.g., eating, workout, substance use, expressive suppression, or aggression). Some ER strategies primarily rely on intrinsic or intrapersonal processes (e.g., distraction, expressive suppression), and some rely primarily on extrinsic or interpersonal processes, that is, regulation through social engagement with others (e.g., seeking consolation, social modeling; Hofmann et al., 2016; Phillips & Power, 2007). Historically, intrinsic ER have been overemphasized in ER research (McRae & Gross, 2020), and further research into understanding the development of these intrinsic ER strategies across development is undoubtedly important. Nevertheless, regardless of age, we often turn to others for help with regulating our emotions, and research on the use of such interpersonal ER strategies has seen increasing research attention in recent years (e.g., Dixon-Gordon et al., 2015; Marroquín, 2011; Nozaki & Mikolajczak, 2020; Zaki & Williams, 2013). In the AERSQ, at least one subscale falls within each of these categorizations.

In addition, ER strategies can be categorized as adaptive or maladaptive, in the sense that they are either effective or ineffective in modifying emotions and/or they are associated with either long-term negative or positive outcomes (Aldao et al., 2010). Some ER strategies are broadly characterized as maladaptive (e.g., expressive suppression or experiential avoidance) and others as adaptive (e.g., acceptance or reappraisal). Although evidence supports such a distinction at least as a useful heuristic (e.g., Schäfer et al., 2016), it is important to note that no strategy is inherently one or the other. Instead, adaptive ER is arguably best understood as the appropriate and effective use of ER strategies considering the context, which can include specific aspects of the culture one finds oneself in, as well as personal goals both immediate and long-term (Aldao, 2013). Conversely, maladaptive ER or emotion dysregulation can either imply a lack of successful regulatory action (e.g., rumination) or the inappropriate and ineffective use of ER strategies in considering personal goals (e.g., aggressive behavior such as lashing out at someone, despite valuing their friendship). For example, distraction has an ambiguous status in that when combined with an attitude of acceptance rather than avoidance, distraction can be supportive of positive psychological development (Wolgast & Lundh, 2017). The AERSQ aims to cover dimensions that are typically viewed as both maladaptive and adaptive.

## Emotion Regulation Development and Psychopathology in Adolescence

Developmental research suggests that children initially rely on caregivers to regulate their emotions and that they gradually come to internalize their ER abilities across early childhood (Eisenberg et al., 1998; Kopp & Neufeld, 2003). These abilities further develop as children reach adolescence (Zeman et al., 2006), suggesting that as children mature, they gradually become more skillful at regulating their own emotions. Despite this, adolescence is a period characterized by greater emotional instability and negative affectivity (Larson et al., 2002), and many psychopathologies implicating ER tend to emerge during adolescence (Kessler et al., 2005; F. S. Lee et al., 2014; Paus et al., 2008).

Some studies have highlighted important differences in ER strategy use that characterize adolescence as compared with other age groups. Cracco and colleagues (2017) looked at the frequency of use for seven typically adaptive and five typically maladaptive ER strategies and found evidence suggesting that in adolescence there is a shift toward more frequent use of maladaptive ER strategies. Zimmermann and Iwanski (2014) looked at adolescents’ and adults’ subjective beliefs about their regulation of negative emotions such as anger, fear, and sadness. They found that individuals aged 13 to 15 report having a smaller repertoire of ER strategies at their disposal than both preceding and succeeding age groups. The combination of a general shift toward more maladaptive types of ER with a general reduction of the ER strategy repertoire available suggest a particular vulnerability with regard to emotional symptoms during adolescence that can in part be accounted for by changes in ER during the period.

The social reinforcement of ER is an additional, potentially interacting, factor, as the use of many ER strategies is necessarily linked to a social context. Cultural factors, for instance, are likely to have an influence on the adaptiveness of different ER strategies. This includes how expressions of emotion tend to be interpreted differently comparing men and women (Barrett & Bliss-Moreau, 2009), which in turn might reinforce the use of specific regulatory strategies. Correspondingly, studies have documented gender differences in the use of specific ER strategies (e.g., Johnson & Whisman, 2013; Nolen-Hoeksema & Aldao, 2011; Tamres et al., 2002) and linked differences to the prevalence of various psychopathologies such as anxiety, depression, and alcohol abuse (e.g., Bender et al., 2012; Nolen-Hoeksema, 2012). However, the exact role of gender in the link between ER and psychopathology is not entirely understood (for a discussion on the subject, see Nolen-Hoeksema, 2012).

## Measuring ER in Adolescence and the Rationale Behind the AERSQ

Given the importance of ER in adolescent psychopathology, it is important that we develop valid measures of ER specifically tailored toward adolescents. In a review by Adrian and colleagues (2011) that examines the assessment of ER, it was estimated that 44% of ER studies that focused on middle childhood (age 6–12) deployed self-report measures, whereas 92.6% of studies that focused on adolescents (age 13–18) deployed self-report measures. Clearly, self-report has become the method of choice in measuring ER among adolescents. However, a large portion of these studies used self-report instruments originally developed for children or adults. This is problematic because adolescence differs from both earlier childhood and adulthood across a range of cognitive and self-evaluative capabilities, which suggest that the same set of questions may be interpreted differently by adolescents than the intended age group (Zeman et al., 2007). For instance, the Emotion Regulation Questionnaire (ERQ; Gross & John, 2003) was originally developed for adults and adapted for use with children and adolescents by Gullone and Taffe (2012). It includes items such as “When I’m worried about something, I make myself think about it in a way that helps me feel better,” which require relatively elaborate self-evaluative knowledge about the interrelation between cognition, emotion, and behavior. Although difficult in practice, it is crucial that measures tailored for adolescents reduce such complex meta-cognitive requirements to a minimum, as such capabilities may be insufficiently developed (Casey et al., 2008). At the same time, adolescents, compared to younger children, tend to exhibit advancements in areas such as abstract and hypothetical thinking, logical reasoning, and information-processing efficiency (Steinberg, 2005). Therefore, it is appropriate to expect them to handle at least moderately more complex inquiries. To achieve the fine balance that meet adolescents at their own level, we must design instruments specifically tailored to their unique set of cognitive and metacognitive abilities, which has been a key focus of the AERSQ.

In addition, most of the work on ER both in adults and in young samples emphasize intrinsic ER strategies (McRae & Gross, 2020). This can be seen in that many of the most popular self-report instruments today only assess intrapersonal ER strategies, such as the ERQ, the Difficulties in Emotion Regulation Scale (DERS; Gratz & Roemer, 2004), and the Cognitive Emotion Regulation Questionnaire (CERQ; Garnefski et al., 2001). An exception is the Interpersonal Emotion Regulation Questionnaire (IERSQ; Hofmann et al., 2016), which explicitly target extrinsic ER. However, the IERSQ was not developed with adolescents specifically in mind. Moreover, it only measures extrinsic ER and must therefore be combined with scales targeting intrinsic ER (e.g., the DERS and CERQ) for an encompassing assessment of ER. The Regulation of Emotion Questionnaire (REQ; Phillips & Power, 2007) and FEEL-KJ (Grob & Smolenski, 2005) are perhaps the most comprehensive alternatives that have been specifically developed for adolescents. REQ encompasses four subscales dividing ER strategies into internal-functional, internal-dysfunctional, external-functional, and external-dysfunctional. However, as previously discussed, the functionality of ER strategies is partly dependent on contextual factors rather than the type of ER strategy alone (Aldao, 2013; Bonanno & Burton, 2013), and the AERSQ, in contrast to REQ, aims to delineate between strategies based on their presumed underlying processes rather than their presumed functionality. In addition, the REQ is yet to be extensively validated, with the original sample only consisting of 225 adolescents (12-19 years) with exploratory and confirmatory factor analyses performed on the same sample (Phillips & Power, 2007). FEEL-KJ is a comprehensive measure comprising 12 subscales that assess 15 ER strategies. Although showing promising psychometric properties in a validation study (Cracco et al., 2015), it is a lengthier instrument containing 90 items. In contrast, the AERSQ encompasses a wide range of ER types while maintaining a succinct format. This can be beneficial to projects that encompass a wide array of different questionnaires, where brevity is an important consideration.

Finally, although some ER measures show good psychometric properties when applied to adolescent samples, the number of strategies assessed across these measures is limited. For instance, the ERQ only assesses positive reappraisal and expressive suppression (Gross & John, 2003). The CERQ, although developed for adolescents, focuses on cognitive ER strategies such as acceptance, rumination, catastrophizing, and positive refocusing (Garnefski et al., 2001). The AERSQ encompasses dimensions of ER captured by some of these instruments (e.g., rumination, positive reorientation, distraction) but also includes at least one dimension of ER not assessed by other instruments that we are aware of, namely, the use of expressive/creative behaviors (e.g., writing down thoughts about felt emotions) to cope with negative affect (assessed by the subscale previously called “cultural activities,” here renamed “creative expression”). Across the different ER strategy measures, it is more common to include inhibitions of emotional expression (e.g., Gross & John, 2003; Hofmann & Kashdan, 2010; Zeman et al., 2001), and few instruments focus on actualized expression of emotions. Art-based activities have previously been linked to positive mental health and to ER in particular (e.g., Geipel et al., 2018; Saarikallio, 2010; van Lith et al., 2013; Zhao et al., 2016), making it an important factor to consider.

In a further developed version of the AERSQ, we aimed to add a second expressive subscale intending to capture aggression as an ER strategy. This inclusion was motivated in several ways. First, when reanalyzing data from earlier iterations of the AERSQ, such a factor emerged in the larger item pool. Second, aggression as an ER strategy has been successfully included in some previous ER instruments targeting adolescents such as FEEL-KJ (Grob & Smolenski, 2005) and REQ (Phillips & Power, 2007), suggesting its relevance to the target age group. Third, the use of aggression to regulate affect was among the maladaptive ER strategies that peaked during adolescence in the study by Cracco and colleagues (2017), implying that it is an especially relevant measure to capture when studying adolescent ER. Finally, previous studies have suggested that aggressive tendencies in adolescence—including experiencing or regulating emotions such as anger toward oneself, self-hatred, or anger toward others—is associated with engaging in NSSI (Boxer, 2010; Brunner et al., 2007; Daukantaitė et al., 2019; Fliege et al., 2009; Sourander et al., 2006; Tang et al., 2013), making it an especially important measure for those who self-injure, with NSSI showing the highest rates of lifetime prevalence during adolescence (Swannell et al., 2014).

## The Current Study

In this study, we aim to evaluate the psychometric properties (factor structure, internal consistency, and construct validity) of a modified and extended version of the AERSQ, referred to as the *Adolescents’ Emotion Regulation Strategies Questionnaire—Extended* (AERSQ-E). As part of the validation process, we also intended to establish the convergent and discriminant validity of the AERSQ-E by exploring the associations between adolescents’ ER strategies and NSSI, DE, as well as other aspects of mental health including internalizing problems (e.g., emotional symptoms, depression, and anxiety), externalizing problems (e.g., peer problems and conduct problems), and positive functioning (life satisfaction). We predicted that the associations for subscales existing in the previous version would align with those previously found (see Zhou et al., 2020). That is, we expected rumination/negative thinking to be positively associated with constructs related to negative functioning and negatively associated with indicators of positive functioning (i.e., life satisfaction), while we expected the opposite pattern for positive reorientation and social support. Furthermore, we expected the creative expression subscale and the distraction subscale to only show weak or no associations to other variables. Finally, for the new theoretical subscale aiming to capture aggression as an ER strategy, we expected to find positive associations to most negative constructs including NSSI, anxiety, and depression, as well as various psychological difficulties including conduct, emotion, and peer problems, which would corroborate the status of aggression as a maladaptive type of ER and the previously discussed evidence linking it to engagement with NSSI.

## Method

### Short Overview

The AERSQ presents respondents with a list of possible behaviors and ways of thinking and asks respondents to judge on a 5-point scale ranging from 1 (*never*) to 5 (*very often*) how often they engage in each item whenever they feel “sad, disappointed, nervous, afraid, or experience other negative or distressing feelings.” Item development of the original measure described in a recent article by Zhou et al. (2020) combined a theory-driven approach with feedback given from a pilot sample allowing adolescents to provide their own examples of ER strategies used. This resulted in a final 25-item version identifying five factors, including rumination/negative thinking, positive reorientation, communication, distraction, and cultural activities.

Based on the original AERSQ scale and new theoretical considerations, an extended and modified version consisting of 33 items was first generated. Specific to this extended version, we generated five items aiming to capture a theoretical construct termed aggressive outlet, reflecting the tendency to regulate emotions using forms of aggression. We also modified the item list of each subscale to improve the conceptual separation of factors and in some cases, the interpretability of the items. This led to a relatively large change to the overall item list for the new version compared with the old one (a lengthier discussion of this procedure including motivation for each item change can be found in the supplementary material under “Revising the scale”). During this process, some factors were reinterpreted and given new names so that the terminology would more closely reflect the presumed underlying function. For instance, the factor previously called cultural activities was renamed creative expression, and the factor previously called communication was renamed social support (for a full comparison between versions including changes to the item pool, see Supplemental Table S1).

In total, six different samples (1,104 students in total, aged 12–20 years; a more detailed description of the samples is provided in the next section) were used to develop and validate the extended version of the AERSQ in the present study. We used Sample 1 and Sample 2 (age ranging from 12 to 16, M_age_ = 14.15) to refine and narrow down the first version of the AERSQ-E to a set of 20 items distributed across five factors (four items per factor) based on interitem correlations as well as each item’s theoretical meaningfulness and distinctiveness as an indicator of a particular factor. These five factors included rumination/negative thinking, positive reorientation, creative expression, aggressive outlet, and social support. However, it became evident that the factor aiming to assess distraction still needed further revising. Consequently, in Sample 3, improvements were made specifically for this subscale, and we managed to narrow down a final distraction subscale consisting of three items. This resulted in a final version consisting of 23 items in total.

In the remaining three samples (4, 5, and 6), the psychometric properties of the final version of the AERSQ-E were evaluated. This validation took place both among adolescents enrolled in junior high school (age ranging from 13 to 17, M_age_ = 14.42; Samples 4 and 5) and high school (age ranging from 16 to 20, M_age_ = 17.10; Sample 6), in total covering an age range from 13 to 20 years. For external validity analyses, data from Sample 2 and Sample 3 were also included wherever data were available.

### Respondents and Recruitment

Data came from samples spread across several schools and municipalities in the southern part of Sweden. According to data acquired from Statistics Sweden dated December 31, 2021, the average income of each municipality’s adult population ranged from below to above the Swedish average (239,664 SEK/year vs. sample range 218,553–286,105 SEK/year). Adult education level was similar to or higher than Sweden as a whole (46% vs. sample range 44%–65% having a university education). The municipalities in which data were collected were slightly more urban (87.6% vs. sample range 90.9%–96.6% living in urban areas).

The data collection spanned two separate projects: one focused on self-harm in adolescence and one focused on self-control during adolescence. Thus, data were collected across several samples of Swedish adolescents aged 12 to 20. All participants responded to at least one version of the AERSQ-E and provided information about their gender and age. As the data were collected within two separate larger projects, additional psychological variables were available for some but not all samples. These include measures of psychological difficulties, depression and anxiety, life satisfaction, non-suicidal self-injury, and DE (see “Measures” section below for more details).

#### Sample 1—Private Junior High School—Initial AERSQ-E Version

The first sample comprised 254 adolescents (130 girls, 121 boys, 18 undisclosed or not identifying as either a girl or boy) in seventh to ninth grade. Ages ranged from 12 to 16 (mean [*SD*] age = 14.17 [0.96], missing = 13). Data were collected during a separate lecture hour and administered alone by teachers at the school provided with a link to the survey by the researchers. Since respondents of this sample were only asked to respond to the AERSQ-E and not measures directly associated with psychological distress (e.g., measures of self-injury and depression), clinical psychologists were available by phone or e-mail rather than in person.

#### Sample 2—Public Junior High School—Initial AERSQ-E Version—Self-Harm Project

The second sample comprised 232 adolescents (102 girls, 126 boys, 4 undisclosed or not identifying as either a girl or boy) in Grades 7 to 9. Ages ranged from 13 to 16 (mean [*SD*] age = 14.13 [0.88]). The survey was administered by a clinically trained researcher along with a research assistant, two master’s students, and teachers at the school. The collection was conducted during a separate lecture hour in a school classroom.

Test–retest data also became available for 178 respondents (mean age [*SD*] = 14.20 [0.86], 76 girls, 99 boys, 3 undisclosed or not identifying as either a girl or boy) from this sample at a delay ranging from 28 to 35 days (response rate = 76.6%). Students were provided digital access by e-mail, and the scale was administered by teachers at the school. The retest comprised only the AERSQ-E and one other scale that assesses embodiment, which was not part of this study.

#### Sample 3—Public Junior High School—Second AERSQ-E Version —Self-Harm Project

The third sample comprised 280 adolescents (151 girls, 126 boys, 3 undisclosed or not identifying as either a girl or boy) in Grades 7 to 9. Ages ranged from 13 to 16 (mean [*SD*] age 14.17 [0.85]). The survey was administered by a clinically trained researcher along with a research assistant, two master’s students, and teachers at the school. The collection was conducted during a separate lecture hour in a school classroom.

#### Sample 4—Public Junior High Schools—Final AERSQ-E Version—Self-Harm Project

The fourth sample comprised 107 adolescents (51 girls, 57 boys, 2 undisclosed or not identifying as either a girl or boy) in Grades 7 to 9. Ages ranged from 13 to 17 (mean (*SD* age 14.37 [0.96]). The survey was administered by a clinically trained researcher and teacher at the schools. The collection was conducted during a separate lecture hour in a school classroom. Sample 4 and 5 form a combined sample for the validation of the AERSQ-E in Grade 7 to 9 but differ in terms of other measured variables.

#### Sample 5—Public Junior High School–Final AERSQ-E Version—Self-Control Project

The fifth sample comprised 145 adolescents (74 girls, 70 boys, 1 undisclosed or not identifying as either a girl or boy) in Grades 7 to 9 across two junior high schools. Ages ranged from 13 to 16 (mean [*SD*] age 14.45 [0.97]). The survey was administered by a clinically trained researcher, a research assistant, and teachers at the schools. The collection was conducted during a separate lecture hour in a school classroom. Sample 4 and 5 form a combined sample for the validation of AERSQ-E in Grade 7 to 9 but differ in terms of other measured variables.

#### Sample 6—Two Private High Schools–Final AERSQ Version—Self-Control Project

The sixth sample comprised 340 adolescents (170 girls, 166 boys, 6 undisclosed or not identifying as either a girl or boy) enrolled in two private high schools in southern Sweden. Ages ranged from 16 to 20 (mean [*SD*] age 17.10 [0.91], missing = 25). The survey was administered by a clinically trained researcher and a research assistant. The collection was conducted either during a separate lecture hour in a school classroom or using a digital classroom in Microsoft Teams.

### Procedure

Respondents in all samples answered the survey digitally using either personal or school-distributed laptops, tablets, or cell phones. All respondents were informed of the purpose and contents of the study, that personal information (which was kept as part of the longitudinal aspirations of the broader project) would not be part of the analysis, and that participation was voluntary. At least one clinically trained psychologist was available either on site or/and by phone or e-mail to handle any iatrogenic effects or other problems and concerns that could arise during or after the administration of the questionnaire (see specific samples above for more details). All respondents provided consent prior to participation, and for all respondents under the age of 15, parental consent was obtained as well in accordance with Swedish law. Ethical approval was provided by the Swedish national ethics review board (registration numbers 2020-05885; 2021-06695-01; 2022-02093-02).

### Measures

Across the different samples, we collected several psychological variables used to assess the convergent and discriminant validity of the AERSQ-E. Reliability estimates and an overview of which variables were collected in each sample of the current study are presented in Table 1.

**Table 1:** Internal Consistency Coefficients (Cronbach’s α) for Variables Used for Evaluation of Convergent and Discriminant Validity of the AERSQ-E Across Samples

Scale	Sample 2 (*n* = 236–237)	Sample 3 (*n* = 281–283)	Sample 4 + 5 (*n* = 250–252)	Sample 6 (*n* = 350–351)	Combined (*n* = 1,017–1,023)
SDQ-s					
Hyperactivity	.78	.78	.82	.75	.78
Conduct	.63	.55	.55	.46	.56
Emotion	.77	.75	.77	.72	.74
Peer	.48	.63	.60	.57	.59
RCADS-25					
Anxiety	.87	.87	.88	.86	.86
Depression	.86	.89	.88	.87	.88
SLSS	.88	.92	.90	.87	.90
DSHI-9r	.90	.88	.90^a^	—	.88
RIBED-8	.88	.88	.87^a^	—	.88

*Note.* SDQ-s = Strength and Difficulties Questionnaire–self-report version; RCADS-25 = Revised Child Anxiety and Depression Scale–shortened version; SLSS = Students’ Life Satisfaction Scale; DSHI-9r = Deliberate Self-Harm Inventory–9-item version; RIBED = Risk Behaviour related to Eating Disorders; AERSQ-E = Adolescents’ Emotion Regulation Strategies Questionnaire–Extended.

a Measured only in sample 5 (*n* = 107).

*Psychological difficulties* were assessed using the Strengths and Difficulties Questionnaire–self-report version (SDQ-s; Goodman, 1997). We used four subscales consisting of five items each: hyperactivity/inattention (e.g., “I am easily distracted, I find it difficult to concentrate”), emotional symptoms (e.g., “I worry a lot”), conduct problems (e.g., “I get very angry and often lose my temper”), and peer relationship problems (e.g., “Other children or young people pick on me or bully me”). Items are rated on a 3-point scale (0 = *not true*, 1 = *somewhat true*, 2 = *certainly true*) taking the past 6 months into consideration. The Swedish version was empirically validated by Lundh et al. (2008) where it was shown to have similar psychometric properties to other language versions.

*Depression and anxiety* were assessed using a short version of the Revised Children’s Anxiety and Depression Scale (RCADS; Chorpita et al., 2000). The shortened 25-item version (RCADS-25) comprises two subscales aiming to measure anxiety and depression and has been shown to have comparable psychometric properties to the long version, with alpha values ranging from .86 to .91 for the anxiety subscale and .79 to .80 for the depression subscale (Ebesutani et al., 2012). The anxiety subscale consists of 15 items (e.g., “I worry when I think I have done poorly at something”) and the depression subscale consists of 10 items (e.g., “Nothing is much fun anymore”). Items are rated on a 4-point Likert-type scale ranging from 0 (*never*) to 3 (*always*) where high scores represent high degrees of anxiety or depression, respectively.

*Life satisfaction* was assessed using the Student’s Life Satisfaction Scale (SLSS; (Huebner, 1991a, 1991b). The scale consists of six items (e.g., “My life is going well”) rated on a 6-point scale ranging from 1 (*strongly disagree*) to 6 (*strongly agree*). The original article (Huebner, 1991a) demonstrated good test–retest reliability and α values of .80 and .82 across two studies.

*Non-suicidal self-injury* was assessed with a modified version of the Deliberate Self-Harm Inventory (DSHI; Gratz, 2001) that was shortened to a nine-item version (DSHI-9r) and adapted to Swedish adolescents in previous research where it showed good test–retest reliability and α values ranging from .66 to .90 (Bjärehed & Lundh, 2008; Lundh et al., 2007; Lundh et al., 2011). The scale asks respondents to use a Likert-type scale from 0 (*never*) to 6 (*more than five times*) to indicate how often they had deliberately injured themselves (e.g., by cutting, carving, or severely scratching themselves, or preventing wounds from healing) in the past 6 months. All individual items were summed into a total score (ranging 0–54) reflecting frequency of engagement.

*Disordered eating* was assessed using the eight-item scale Risk Behaviour related to Eating Disorders (RiBED-8; Waaddegaard et al., 2003). The scale was developed as a screening instrument aimed at assessing the prevalence of risk behaviors associated with eating disorders. Respondents are asked to rate on a 4-point scale ranging from 1 (*almost never/never*) to 4 (*very often*) how often they engage in thoughts or behaviors associated with food consumption (e.g., “I vomit to rid myself of food I have eaten”). In their original paper, Waaddegaard et al. (2003) demonstrated that the RiBED had good test–retest reliability and ability to identify persons with eating pathology. In previous studies targeting Swedish adolescents, alpha values have ranged from .74 to .78 (Bjärehed & Lundh, 2008; Lundh et al., 2008).

### Data Analyses

#### Missingness and Exclusion

The missing completely at random (MCAR) assumption was rejected by Little’s MCAR test for Sample 1, χ^2^(912) = 1,017.47, *p* = .008, Sample 3, χ^2^(4,580) = 4,962.04, *p* < .001), and Sample 5, χ^2^(2,534) = 2,712.51, *p* = .007. However, in all three samples, the ratio of χ^2^ to *df* was below 2, suggesting that the deviance from the MCAR assumption was minor (Ullman & Bentler, 2013). In addition, all items had relatively low percentage totals missing (range across samples: 1.4–5.9%). Finally, inspection of the multivariate missing patterns using *MICE* package in R (van Buuren & Groothuis-Oudshoorn, 2011) showed that no instances of multiple missing data occurred more than once. These patterns of missingness were therefore presumed as not meaningfully different from MCAR in any sample. Accordingly, prior to conducting any analyses, we listwise deleted 12 systematic responders because they had zero variance in the AERSQ-E, and 42 respondents missing more than 10 % in the AERSQ-E, suggesting that imputing their data could significantly bias the factor analyses.

The remaining missing data values were imputed in one of two ways depending on the measure. Missing items in the RIBED-8 and DSHI-9r, both of which tend to be positively skewed, were imputed with 0 for all cases with less than 3 missing values as is common practice (e.g., Lundh et al., 2008; Viborg et al., 2014). All other missing values were imputed using the expectation–maximization (EM) algorithm from the *mvdalab* package in R (Afanador et al., 2021). Items were imputed at item-level with other values as predictors, apart from five respondents who did not respond to some scales in full and consequently had their data for those scales imputed at the scale-level using the other scales are predictors. All analyses reported in the current paper were run with both the imputed and the nonimputed data, and since imputations did not markedly change any of the results, all results presented below are based on the imputed data.

#### Measure Development

For Samples 1 and 2 and later Sample 3, through which the final version was generated, exploratory factor analyses (EFA) on the AERSQ-E utilized an ordinary least squares solution with oblimin, an oblique rotation method which assumes that the latent variables can be correlated. The optimal number of factors was determined using parallel analysis, which compares the scree of eigenvalues of the observed data with a Monte Carlo–simulated matrix of data of the same size. We also considered which factors had an eigenvalue >1 and the point of inflection in the scree plot. All analyses were conducted with the *psych* package in R (Revelle, 2017). We initially opted to retain a maximum of four items in each subscale with factor loadings above .4. We also aimed to identify an item pool with satisfactory coverage of each identified ER strategy and thus screened for items too highly correlated.

#### Measure Testing

Confirmatory factors analysis (CFA) was used to evaluate the suggested EFA structure of the AERSQ-E in Samples 4 to 6. The CFA was run with a maximum likelihood (ML) estimator using the lavaan package in R (version 0.6.9, Rosseel, 2012). Model fit was evaluated using the root mean square error of approximation (RMSEA) with 95 % confidence intervals, the Comparative Fit Index (CFI), and the standardized root mean residual (SRMR). An acceptable model fit was defined as CFI > .90, RMSEA < .06, and SRMR < .09, and a good model fit was defined as CFI > .95, RMSEA < .05 and SRMR < .08 (Hooper et al., 2007; Hu & Bentler, 1999). In case of a poor model fit, potential improvements to the models were also evaluated using modification indices and the theoretical soundness of the proposed additional covariances not implied by the original EFA.

The best-fitting AERSQ-E models were subsequently tested for measurement invariance (MI) across age and gender (S. T. Lee, 2018; Milfont & Fischer, 2010). Testing for MI requires sufficiently large test groups that are of about equal size (Chen, 2007). When using RMSEA as a fit index, it has been suggested that *n*>100 is recommended for correct rejection of models (Putnick & Bornstein, 2016). As the sample size was below 100 for most specific ages, we opted to assess age by comparing adolescents attending junior high school (Samples 4–5, *N =* 252, age range 13–17, mean age = 14.41) to those attending high school (Sample 6, *N* = 340, age range 16–20, mean age = 17.10). For model comparison, we relied on CFI, RMSEA, and SRMR as has become common practice (Putnick & Bornstein, 2016) due to the sensitivity of chi-square difference tests to sample size and deviations from normality assumptions (Chen, 2007; Sass, 2016). Here, we considered values of ΔCFI < .01, ΔRMSEA < .015, and ΔSRMR < .03 as indicating sufficient model similarity to conclude metric invariance, and ΔCFI < .01, ΔRMSEA < .015, and ΔSRMR < .01 scalar to conclude scalar invariance (Chen, 2007; Sass, 2016).

Next, the implied subscales of the AERSQ-E were tested for internal consistency, test–retest reliability, and divergent or discriminant validity. Internal consistency was assessed using Cronbach’s α values, where we sought an α of .7 or above. The test–retest reliability coefficients were computed for all but the distraction subscale using data from Sample 2. We calculated correlations between the AERSQ-E subscales and measures of NSSI, DE, internalizing/externalizing problems, emotional distress, and positive functioning to evaluate convergent and discriminant validity. Correlations are presented using Pearson’s *r* for ease of interpretability as nonparametric alternatives yielded comparable results in terms of statistical significance and effect size when the assumptions were violated. Bonferroni-corrected significance values were used to avoid spurious correlations and applied across all parametric tests, where we used a corrected alpha of .05/69 = 0.0007.

## Results

### Exploratory Factor Analysis

Parallel analysis with data from Sample 1 and Sample 2 on the initial version of the AERSQ-E suggested six factors to best fit the data. The same was suggested by looking at eigenvalues and the point of inflection in the scree plot. Based on EFA, four items were retained per subscale (for a summary of the results from this factor analysis, see Table S2 of the Supplementary). However, poor factor loadings for many items included in the distraction subscale suggested it required further revision. After a close inspection of the items, we theorized that the poor psychometric properties of the distraction subscale were likely caused by the specificity of the items, in that, compared with other subscales, it contained much more specific activities putatively reflecting a distraction-oriented ER. Examples include “Watch something (e.g., a movie, television shows, streaming)” or “Play games (e.g., video or computer games).” These activities, although presumably reflecting distractive activities, might not be endorsed within-person consistently such that a person might prefer one distractive activity over all others. Although in line with the focus of the AERSQ-E on assessing specific ER behaviors, we decided to reformulate the scale to focus more on the cognitive processes at work, hopefully allowing the subscale to identify distraction more consistently and independently as an ER strategy across all items. Examples of new items include “Try to think about something else” and “Distract myself with something to do” (also see Table 2).

**Table 2:** Exploratory Factor Loadings for the Final Version of the AERSQ-E (Sample 3)

Item (in English translation)	Sample 3					
	Factor					
	1	2	3	4	5	6
R1. Think about things I have said or done (again and again)	**.60**	–.05	.17	–.09	.00	.06
R2. Think that I am bad or worthless	**.69**	–.25	.05	–.02	–.01	.04
R3. Worry about what others might think of me	**.66**	–.10	.03	–.10	.02	–.02
R4. Believe that others have it much better off than me	**.64**	.02	–.05	.16	.02	.01
P1. Try to find something positive in what has happened	–.01	**.56**	.04	.01	.06	.16
P2. Move on and try to do things better next time	–.03	**.73**	.00	–.13	–.02	.05
P3. Try in a calm manner to solve what made me feel bad	.09	**.32** ^a^	.12	–.15	.28	.21
P4. Stay calm and think that it will pass	–.10	**.81**	.01	.00	.03	–.01
C1. Create something that expresses how I feel	–.19	–.04	**.71**	.07	.02	.11
C2. Write texts (e.g., stories, song lyrics, poems)	.15	.10	**.64**	.00	–.04	–.11
C3. Draw or paint	–.02	–.01	**.64**	.02	.01	.02
C4. Write down thoughts about how I feel	.27	.05	**.58**	–.04	.02	–.06
A1. Punch or kick on things	–.13	–.09	.00	**.71**	–.08	–.02
A2. Try to find something to break	–.06	–.10	.04	**.74**	.02	.04
A3. Argue or fight with people around me	.40	.00	.02	**.49**	.07	–.05
A4. Want to hurt others (physically or mentally)	.30	.10	.07	**.49**	.00	–.18
S1. Tell someone else how I feel	–.06	–.02	–.10	.01	**.88**	.06
S2. Seek support and comfort in others	.01	.04	.03	.02	**.87**	–.08
S3. Ask others for advice or help	.05	.05	–.02	–.07	**.71**	.01
S4. Seek physical contact (e.g., a hug)	.05	–.06	.28	.00	**.60**	.06
D1. Try to think about something else	.01	.12	–.06	–.01	–.01	**.77**
D2. Distract myself with something to do	–.01	–.11	.12	–.07	.07	**.62**
D3. Try to forget that which makes me feel bad	.01	.07	.01	–.01	–.03	**.57**
*Avoid things that remind me of my feelings* ^b^	.38	.09	–.03	.15	.14	.37
*Pretend like the emotions I feel do not exist* ^b^	.28	.03	.03	.24	–.22	.32

*Note.* The Exploratory Factor Analysis was based on oblimin rotation and extraction by ordinary least squares. Bold represents final factor assignment. AERSQ-E = Adolescents’ Emotion Regulation Strategies Questionnaire–Extended.

a Item P3 was retained with a factor loading of .61 in Samples 1 and 2. ^b^ Items removed from the final version.

A second version of the AERSQ-E, including five new items intended to capture distraction and 20 items retained from the first EFA for the other subscales, was subsequently tested in Sample 3. We limited the second revised distraction subscale to five new items because of a need to minimize the burden put on respondents considering the data collection at large. Once again, parallel analysis again suggested six factors to best fit the data (as did the eigenvalues and point of inflection using a scree plot), and results from the subsequent EFA is presented in Table 2. For the distraction subscale, we selected three items showing satisfactory factor loading and construct coverage, resulting in a final 23-item version of the AERSQ with six different subscales labeled as rumination/negative thinking (4 items), positive reorientation (4 items), creative expression (4 items), aggressive outlet (4 items), social support (4 items), and distraction (3 items). One item from the positive reorientation subscale (i.e., “Try in a calm manner to solve what made me feel bad”) showed low factor loading (*λ*_P_ = .32) as well as cross-loading with aggressive outlet (*λ*_A_ = .28) in Sample 3. However, given the content of the item as well as the previous factor loading from Samples 1 and 2 (*λ*_P_ = .61), we decided to retain this item in the final positive reorientation subscale.

### Confirmatory Factor Analysis

**Figure 1:**
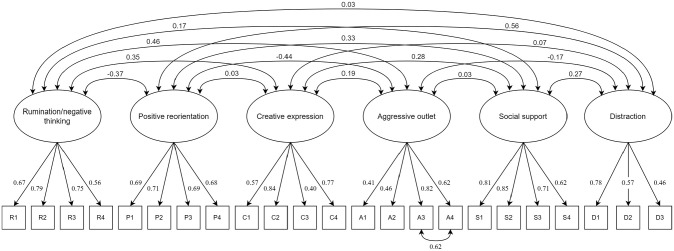
Six Factor AERSQ-E Model With 23 Indicator Items *Note. N* = 592, RMSEA [95% CI] = .053 [.048, .058], SRMR = .061, CFI = .920. Loadings and covariances represent combined data from Samples 4 to 6. See Table S3 of Supplementary for estimated confirmatory factor loadings, covariances and model fit for each sample separately. AERSQ-E = Adolescents’ Emotion Regulation Strategies Questionnaire —Extended; RMSEA = root mean square error of approximation; CI = confidence intervals; CFI = Comparative Fit Index; SRMR = standardized root mean residual.

The final version consisting of 23 items was administered in Samples 4 and 5 (representing respondents in junior high school) and in Sample 6 (representing respondents in high school). When combining all samples (4–6), the six-factor model showed mixed results on fit indices with RMSEA and SRMR showing acceptable values, but CFI was found to be somewhat low (*N* = 592, RMSEA [95% confidence interval, CI] = .064 [.059, .069], SRMR = .066, CFI = .884). However, one pair of items in the aggressive outlet subscale, “I argue or fight with people around me” and “I want to hurt others (physically or mentally)” demonstrated high residual covariance not explained by the latent variable (*σ* = .62). A rerun of the combined sample adding this covariance to the model demonstrated acceptable model fit across all indices (*N* = 592, RMSEA [95% CI] = .053 [.048, .058], SRMR = .061, CFI = .920, modeling these samples separately yielded comparable results, cf. Supplemental Table S3). The loadings and covariances of these models are visualized in Figure 1. The modified model significantly improved model fit (Δχ^2^ = 19.06, *p* < .001).

### Internal Consistency

Table 3 presents the internal consistency values (Cronbach’s α) of the AERSQ for all samples. Across all samples and in combination, alpha values for five out of six factors tended to be in acceptable or high ranges. The exception was a distraction, which showcased adequate values for Sample 3 (α = .71) but somewhat lower values for Samples 4 and 5 (α = .57), Sample 6 (α = .65), and in a pooled analysis (α = .65).

**Table 3:** Cronbach’s α Values for AERSQ-E Subscales Across Samples

Subscale	Sample					
	1	2	3	4–5	6	Combined
Rumination/negative thinking	.80	.84	.79	.80	.77	.80
Positive reorientation	.76	.80	.78	.78	.78	.78
Creative expression	.70	.72	.75	.68	.78	.73
Aggressive outlet	.75	.68	.74	.77	.68	.72
Social support	.82	.88	.85	.79	.84	.84
Distraction	NA	NA	.71	.57	.65	.65_a_

*Note.* AERSQ-E = Adolescents’ Emotion Regulation Strategies Questionnaire–Extended.

a Sample 1 and 2 are not included.

### Test–Retest Reliability

All test–retest correlations were medium to large (*n* = 178): *r* [95% CI] rumination/negative thinking = .77 [.70, .82], positive reorientation = .71 [.62, .77], creative expression = .73 [.66, .79], aggressive outlet .71 [.63, .77], and social support = .77 [.70, .82]. No retest data were available for the distraction subscale, as it was revised in a later sample compared with when test–retest reliability assessments were performed.

### Measurement Invariance

Table 4 summarizes the results for method invariance across age and gender. We found evidence of metric invariance between age groups (i.e., junior high school students were compared to high school students) and between girls and boys across all fit indices of interest (ΔCFI_gender_ = .005, ΔRMSEA_gender_ < .001, ΔSRMR_gender_ = .003; ΔCFI_age_ = .007, ΔRMSEA_age_ = .001, ΔSRMR_age_ = .005). Considering scalar invariance for both age and gender, ΔRMSEA and ΔSRMR suggested scalar invariance but ΔCFI did not (ΔCFI_gender_ = .023, ΔRMSEA_gender_ < .005, ΔSRMR_gender_ = .005; ΔCFI_age_ = .016, ΔRMSEA_age_ = .003, ΔSRMR_age_ = .003). Scalar invariance across all indices (ΔCFI = .008–0.010, ΔRMSEA = .002, ΔSRMR = .002) could only be established by releasing the two items significantly associated with the highest model fit improvement for gender (i.e., “Argue or fight with people around me” and “Seek physical contact (e.g., a hug)”; χ^2^ = 16.44–34.43) and age, respectively (i.e., “Think about things I have said or done (again and again)” and “Distract myself with something to do”; χ^2^ = 19.24–20.28).

**Table 4:** Comparing Configural, Metric and Scalar Invariance Across Gender and Age Groups

Group	Model	χ^2^	*p* value	CFI	RMSEA	SRMR
Gender	1. Configural	806.04	<.001	.909	.055	.061
	2. Metric	835.06	<.001	.906	.055	.064
	|Δ| 2-1	29.02	.034	.003	<.001	.003
	3. Scalar	944.30	<.001	.883	.060	.069
	|Δ| 3-2	109.24	<.001	.023	.005	.005
Age	1. Configural	765.23	<.001	.923	.052	.064
	2. Metric	808.69	<.001	.917	.053	.068
	|Δ| 2-1	43.46	<.001	.007	.001	.005
	3. Scalar	895.94	<.001	.901	.057	.071
	|Δ| 3-2	87.25	<.001	.016	.003	.003

*Note.* RMSEA = root mean square error of approximation; CFI = comparative fit index; SRMR = standardized root mean residual.

### Subscale Intercorrelations

Table 5 presents expected and observed intercorrelations between all subscales of the AERSQ-E. As expected, we found a positive association between rumination/negative thinking and aggressive outlet (Samples 2–6, *r =* .28), and both these were negatively associated with positive reorientation (rumination/negative thinking: Samples 2–6, *r =*–.28; aggressive outlet: Samples 2–6, *r =*–.31). Positive reorientation, conversely, was as expected positively associated with both social support (Samples 2–6, *r* = .27) and distraction (Samples 3–6, *r* = .37). Creative expression was positively associated with social support (Samples 2–6, *r =* .24), rumination/negative thinking (Samples 2–6, *r =* .27), and aggressive outlet (Samples 2–6, *r =* .15), whereas we expected to find a positive association only with social support and distraction. Finally, in line with expectations, social support was weakly but positively associated with distraction (Samples 3-6, *r =* .21). All correlations presented here were significant at *p* < .0007.

**Table 5:** Expected and Observed Intercorrelations Between the AERSQ-E Subscales (Sample 2–6, N = 872–1,104)

Subscale	Expected pattern						Observed pattern						*N*
	R	P	C	A	S	D	R	P	C	A	S	D	
Rumination/negative thinking							1						1,104
Positive reorientation	—						**–.28** ***	1					1,104
Creative expression	0/-	+					**.27** ***	.03	1		.		1,104
Aggressive outlet	++	—	—				**.28** ***	**–.31** ***	**.15** ***	1			1,104
Social support	—	++	+	—			**.11** ***	**.27** ***	**.24** ***	–.04	1		1,104
Distraction	0	++	+	0	+		.06	**.37** ***	.08 *	**–.12** ***	**.21** ***	1	872

*Note.* Significant correlations after Bonferroni correction are shown in bold. Expected patterns reflects the rating of two independent raters +++ = strong positive correlation (*r* > .5), ++ = moderate positive correlation (.3 < *r* > .5), + = weak positive correlation (.1 < *r* > .3), 0 = no correlation, − = weak negative correlation (*r* < –.1), −− = moderate negative correlation (–.1 < *r* > –.3), −−− = strong negative correlation (*r* > –.5). AERSQ-E = Adolescents’ Emotion Regulation Strategies Questionnaire–Extended; Columns R = Rumination/negative thinking; P = Positive reorientation; C = Creative Expression; A = Aggressive outlet; S = Social support; D = Distraction.

* *p* < .05. **p < .01. ***p < .001.

### Convergent and Discriminant Validity

Table 6 presents a summary of results including an expected and observed pattern of association with other psychological variables. Key findings (all significant at *p* < 0007 in accordance with Bonferroni correction) are summarized below.

**Table 6:** Expected and Observed Intercorrelations Between the AERSQ-E and Other Studied Variables in Combined Sample (Sample 2–6, N = 619–1,104)

	Expected pattern						Observed pattern						*N*
Variable	R	P	C	A	S	D	R	P	C	A	S	D	
Psychological difficulties (SDQ)													
Hyperactivity/inattention	+	−	0/−	+	0/−	0	.12 ***	**–.34** ***	.02	**.36** ***	**–.13** ***	–.08	1,104
Peer relationship problems	++	−	0/−	+	−	0	**.17** ***	–.12 ***	**.14** ***	.10 ***	–.04	–.11 **	1,104
Emotional symptoms	+++	0/−	+	+	−	0/+	**.53** ***	**–.29** ***	**.23** ***	**.17** ***	.12 ***	–.01	1,104
Conduct problems	++	−	0	++	−	0	.06 *	**–.26** ***	.04	**.41** ***	**–.14** ***	–.13 ***	1,104
Life satisfaction (SLSS)	–	+	0/+	−	+	0	**–.51** ***	**.42** ***	**–.18** ***	**–.29** ***	**.15** ***	.12 ***	1,104
Anxiety and depression (RCADS-25)													
Anxiety	++	−	NA	+	−	0/+	**.59** ***	**–.27** ***	**.28** ***	**.24** ***	.11 ***	.00	1,104
Depression	+++	−	NA	+	−	0/+	**.51** ***	**–.38** ***	**.20** ***	**.31** ***	–.04	.05	1,104
Self-injury (DSHI)	++	−	NA	++	0/−	0	**.33** ***	**–.38** ***	**.18** ***	**.37** ***	–.08	–.15 *	619
Disordered eating (RIBED-8)	++	−	NA	NA	0/−	−	**.48** ***	**–.33** ***	**.19** ***	**.25** ***	–.01	–.04	619

*Note.* Significant correlations after the Bonferroni correction are shown in bold. Expected patterns reflects the rating of two independent raters (+++ = strong positive correlation (*r* > .5), ++ = moderate positive correlation (.3 < *r* > .5), + = weak positive correlation (.1 < *r* > .3), 0 = no correlation, − = weak negative correlation (*r* < –.1), −− = moderate negative correlation (–.1 < *r* > –.3), −−− = strong negative correlation (*r* > –.5), NA = no predictions made). SDQ = Strengths and Difficulties Questionnaire –self-report version; SLSS = Student’s Life Satisfaction Scale; RCADS = RCADS; DSHI = Deliberate Self-Harm Inventory; RIBED = Risk Behaviour related to Eating Disorders; Columns R = Rumination/negative thinking; P = Positive reorientation; C = Creative Expression; A = Aggressive outlet; S = Social support; D = Distraction.

* *p* < .05. **p < .01. ***p < .001.

As expected, moderate to strong positive associations were found between rumination/negative and negative constructs including anxiety (Samples 2–6, *r* = .59), depression (Samples 2–6, *r* = .51), NSSI (Samples 2–4, *r* = .33), DE (Samples 2–4, *r* = .48), and emotional symptoms (Samples 2–6, *r* = .53). Conversely, rumination/negative thinking had a strong negative correlation with life satisfaction (Samples 2–6, *r* = −.51). We also expected a moderate positive association between rumination/negative thinking and conduct problems, which was not supported by the data.

Similar patterns were observed for an aggressive outlet, which was positively correlated with anxiety (Samples 2–6, *r* = .24), depression (Samples 2–6, *r* = .31), NSSI (Samples 2–4, *r* = .37), DE (Samples 2–4, *r* = .25), and emotional symptoms (Samples 2–6, *r* = .17) and negatively correlated with life satisfaction (Samples 2–6, *r* = −.29). In addition, aggressive outlet had a moderate to strong relationship with hyperactivity/inattention (Samples 2–6, *r* = .36) and conduct problems (Samples 2–6, *r* = .41). However, we did not find the expected positive association between aggressive outlet and peer problems (Samples 2–6, *r* = .10).

Positive reorientation, in line with expectations, was positively correlated to life satisfaction (Samples 2–6, *r* = .42) and negatively correlated to most negative constructs including hyperactivity/inattention (Samples 2–6, *r* = −.34), emotional symptoms (Samples 2–6, *r* = −.29), conduct problems (Samples 2–6, *r* = −.26), anxiety (Samples 2–6, *r* = −.27), depression (Samples 2–6, *r* = −.38), NSSI (Samples 2-4, *r* = −.38) and DE (Samples 2–4, *r* = −.33).

We predicted social support to be weakly but negatively associated with most negative constructs and positively associated with life satisfaction. In line with these predictions, we found a weak but negative correlation between psychological difficulties and social support including hyperactivity/inattention (Samples 2–6, *r* = –.13) and conduct problems (Samples 2–6, *r* = –.14) and a weak positive correlation to life satisfaction (Samples 2–6, *r* = –.15). Although some other weak correlations were also found, these did not retain significance after Bonferroni correction.

For creative expression, we only made predictions for some of the associations, and when we did, we expected weak or no associations. However, we found weak but positive associations to most negative constructs including peer problems (Samples 2–6, *r* = .14), emotional symptoms (Samples 2–6, *r* = .23), anxiety (Samples 2–6, *r* = .28), depression (Samples 2–6, *r* = .20), NSSI (Samples 2–4, *r* = .18), and DE (Samples 2–4, *r* = .19) and weak but negative association to life satisfaction (Samples 2–6, *r* = −.18).

Finally, given the ambiguous functionality of distraction as an ER strategy, we expected its subscale to have few and weak associations with other measured constructs. This was confirmed in that all correlations were weak, and none retained significance after the Bonferroni correction.

## Discussion

In this study, we presented the psychometric properties of the AERSQ-E, a modified and extended version of the original AERSQ developed by Zhou and colleagues (2020). Across six community samples of Swedish youth (aged 12–20), we generated and narrowed down a final 23 item version of the AERSQ-E and evaluated its internal structure, reliability, and validity as a measure assessing the use of different ER strategies in adolescence.

The factor analyses generally supported a six-factor structure for the AERSQ-E including rumination/negative thinking (4 items), positive reorientation (4 items), creative expression (4 items), aggressive outlet (4 items), social support (4 items), and distraction (3 items). These factors showed low to moderate correlations to each other. Using confirmatory factor analysis, both SRMR and RMSEA showed adequate model fit for the AERSQ-E. However, the CFI did not reach adequate values unless the residual of two aggressive outlet-related items (i.e., “Punch or kick things” and “Try to find something to break”) was covaried as suggested by the modification indices. Post hoc modifications of the factor structure of a measurement should only be pursued when empirically or conceptually justified (MacCallum et al., 1992). In this case, the two items for which residuals were covaried reflected tendencies to direct aggressive behaviors toward inanimate objects, while the other two items of the aggressive outlet subscale reflected aggression aimed at other people. We deemed this modification to be justified although it was applied post hoc, where we reasoned that the conceptual delineation this modification reflected between aggression toward inanimate objects or toward other people (i.e., socially) does not take away from the use of the subscale as a measure of ER through aggression at a more general level.

Assessing measurement invariance, we found that metric invariance of the modified factor structure of the AERSQ-E was supported across girls and boys and in comparing junior high school students and standard high school students. However, while the ΔRMSEA and ΔSRMR gave support to scalar invariance across these gender and age groups, the ΔCFI did not. We note that there are currently no clear conventions on how to interpret inconsistent results across different fit indices, and there is large variability in cut-off levels as well as what fit indices should be considered most important in evaluating measurement invariance (see Putnick & Bornstein, 2016, for a discussion on conventions of measurement invariance testing). Nevertheless, scalar invariance is important because it ensures that statistical differences in group means reflect actual differences in ER strategy preferences and not unintended, biasing properties of the scale (S. T. Lee, 2018). We managed to establish partial scalar invariance by releasing some items but have no strong theoretical reasons behind these specific modifications. Future work could examine more closely why specifically these items are interpreted differently by girls and boys and by different levels of high school students. Some potentially important factors are the role played by social reinforcement in emotion and ER (e.g., Barrett & Bliss-Moreau, 2009; Nolen-Hoeksema, 2012) as well as levels of cognitive maturation (e.g., Ahmed et al., 2015; Casey et al., 2008).

Regarding internal consistency, we found that all subscales except distraction had acceptable internal consistency (i.e., α > .70) and good test–retest reliability (i.e., *r* > .70). Alpha values for the distraction subscale varied between 0.57 and 0.71 across different samples. In a recent review on the use of Cronbach’s α in research on instrument development, Taber (2018) provided illustrative examples from the science education literature showing a wide range of alpha values being treated as acceptable or satisfactory (e.g., as low as α = .45). The article raised concerns with the arbitrary value of .70 as a sufficient measure of acceptable internal consistency, citing several influential statisticians. For instance, although Cronbach (1951) himself suggested that a high value of alpha was “desirable,” he also emphasized the importance of instrument interpretability which, according to him, was often possible without having high values of alpha. Similar conclusions have been made by Schmitt (1996) claiming there is no general level (such as .70) at which α becomes acceptable and that instruments with quite low alpha values can prove useful. In relation to coping and ER specifically, early researchers postulated that a low alpha value is sometimes expected if the use of one type of coping (ER) strategy obviates the use of another (Billings & Moos, 1981). Given that the distraction subscale comprised only three items, meaning that each item intercorrelation has a considerable effect on the α value (the average inter-item correlation for the distraction subscale was *r* = .36), we suggest that a lower-than-usual α value could reflect sufficient construct coverage (Taber, 2018) rather than problems of multidimensionality.

With respect to external validity, our findings generally aligned with our expectations. We found that rumination/negative thinking had positive associations with anxiety, depression, DE, and NSSI, aligning with data found using the previous version of the AERSQ (Zhou et al., 2020). Rumination, one of two cognitive strategies assessed by the AERSQ-E and one of the most studied ER strategies, has been extensively linked to several mental health issues, with many studies specifically targeting adolescents (e.g., Calvete et al., 2015; Olatunji et al., 2013; Rood et al., 2009; Royuela-Colomer et al., 2021). Accordingly, we found that rumination/negative thinking had the strongest associations with both positive and negative aspects of mental health and psychological functioning. This aligns with a meta-analytic review by Aldao et al. (2010) who, comparing different ER strategies, suggested that rumination had the strongest effect size in predicting anxiety, depression, eating, and substance-related disorders. Moreover, we found that positive reorientation, the second cognitive strategy assessed by the AERSQ-E, was associated with various aspects of positive functioning, showing positive associations with life satisfaction and negative associations with internalizing and externalizing problems. This corroborates the common understanding of strategies involving positive reappraisal as adaptive (e.g., Cracco et al., 2017; Schäfer et al., 2016) and having wide-ranging benefits to mental health (e.g., Aldao et al., 2010; Nowlan et al., 2015).

Social support, the interpersonal dimension assessed with the AERSQ-E, was weakly but negatively associated with internalizing and externalizing problems and positively associated with life satisfaction. This corroborates findings suggesting that the availability of interpersonal resources can contribute to positive functioning (Dixon-Gordon et al., 2015). However, we urge that future work interprets this scale within context, as who is providing the support could have important implications for the effectiveness and availability of utilizing this strategy to regulate emotion. Contextual interpretation is also necessary for the distraction subscale, as it demonstrated only negligible associations (*r* < .15) with the other variables. Other studies have similarly suggested that distraction is only weakly associated with concurrent and future levels of depression (Rood et al., 2009). These findings reinforce the view that distraction is neither adaptive nor maladaptive outside its context; rather, it can be a predictor of positive functioning when combined with an attitude of acceptance and a predictor of negative functioning when combined with an attitude of avoidance (Wolgast & Lundh, 2017).

Finally, the two expressive ER strategies (i.e., creative expression and aggressive outlet) were in this study both positively related to internalizing and externalizing problems and negatively related to life satisfaction. This is unsurprising for aggressive outlet, as aggression has previously been linked to NSSI and deliberate self-harm (Boxer, 2010; Brunner et al., 2007; Daukantaitė et al., 2019; Fliege et al., 2009; Sourander et al., 2006; Tang et al., 2013) and is largely considered a maladaptive ER strategy (e.g., Cracco et al., 2017; Grob & Smolenski, 2005). Finding consistent, albeit weak, positive links to mental health problems for creative expression was more surprising. In the original AERSQ paper (Zhou et al., 2020), the “cultural activities” subscale (comparable to the creative expression subscale of the current version) produced mixed results, showing positive associations only to NSSI and emotional symptoms, and only at one out of two measured time-points. Conversely, research regarding the influence of performing art-based activities tend to highlight the beneficial effect with regard to mental health (e.g., Geipel et al., 2018; Saarikallio, 2010; van Lith et al., 2013; Zhao et al., 2016), whereas our findings suggest the opposite. It should be noted, however, that the current study is cross-sectional, meaning the associations found here do not necessarily translate to the longitudinal effects of using art-based activities as ER strategies. It is conceivable that there would be prospective beneficial effects of the creative expression subscale when assessed longitudinally except that art-based creative activities are common among a portion of those with a tendency toward issues of mental health (Cropley, 1990). Future longitudinal studies could investigate this further.

An important strength of the present study is that the sample encompassed 1,104 adolescents in total sourced from schools located in Swedish municipalities that were comparable to the Swedish average. In addition, we had roughly an equal number of both adolescent girls and boys covering a wide range of ages. Together, this lends strong support to the representativity of the sample and generalizability of the findings, at least covering a Swedish setting and across countries with comparable demographics. Future studies are needed to evaluate the structure and psychometric properties of the AERSQ-E cross-culturally. Future studies should also examine the instrument among those not identifying as either men/boys or women/girls. Finally, we did not recruit from exclusively clinical populations of adolescents who might show unique difficulties regarding ER, implying that its applicability in a clinical setting has not been established, something we suggest for future studies.

Another strength is that the AERSQ-E encompasses a broad range of important ER dimensions, making it a holistic yet resource-efficient measurement of ER in adolescence. It includes both ER strategies more focused on changes in cognition (i.e., rumination/negative thinking or positive reorientation) and changes in behavior (i.e., creative expression, aggressive outlet, or distraction). The instrument also covers the interpersonal dimension of ER (i.e., social support) contrasting with intrapersonal ER strategies. Moreover, it also captures ER strategies commonly viewed as maladaptive (rumination/negative thinking, aggression outlet) and adaptive (e.g., positive reorientation or social support). Finally, one key feature of the AERSQ is the behavioral ER strategies focusing on expressions of emotions, including the subscales aggressive outlet and creative expression, which is a less frequently considered dimension of ER across measurements, with expressive suppression, i.e., the inhibition of emotional expression, being much more common (e.g., Gross & John, 2003; Hofmann & Kashdan, 2010; Zeman et al., 2001). Thus, the AERSQ-E holds promise to be applicable in a wide range of research projects emphasizing different distinctions in ER.

Beyond the limitations discussed previously, there are a few others that should be discussed. First, given the fact that this study is cross-sectional and correlational, we cannot ascertain whether making use of certain ER strategies poses a risk of developing mental health issues or if it is the other way around, whereby a preexisting level of distress causes the favoring of certain ER strategies over others. For instance, the cross-sectional nature of this study might be the cause of the weak but overall positive association found between creative expression and mental health issues, contrasting previous findings showing mental health benefits for these types of activities (e.g., Geipel et al., 2018; Zhao et al., 2016). Longitudinal assessments are needed to further investigate the validity of each subscale in this regard.

Second, we did not manage to collect test–retest data for the distraction subscale given its late revision in the development process, meaning we could not evidence the longitudinal reliability of this subscale. Given this and its relatively low internal consistency values, future studies are warranted to seek improvements to this subscale in particular.

Third, although our goal was to avoid items requiring complex metacognitive evaluations, the degree to which we managed this is uncertain. For instance, we reformulated the distraction subscale to be more general, thereby increasing its level of abstraction. This means the questions (e.g., “Try to think about something else”) require an ability to not only draw connections between felt emotions and behavior, but also to some extent understand the cognitive processes that drove that behavior. Importantly, these items as well as the items included in the other subscale do not require insight about the consequences of these cognitive and behavioral processes, which would have added metacognitive self-evaluative requirements we deem particularly problematic for assessment in adolescence.

To summarize, the AERSQ-E has several merits as it addresses some limitations posed by the current instruments used to measure adolescent ER, and it showed some improvement in psychometric properties compared with its predecessor, the AERSQ. However, further improvements could be made, and specifically, regarding the distraction subscale the validity and reliability could be further investigated. In addition, the instrument should be validated using longitudinal data and for use with clinical samples. Nevertheless, we believe the AERSQ-E has the potential to contribute to our knowledge of ER across adolescence.

## Supplemental Material

sj-docx-1-asm-10.1177_10731911231164619 – Supplemental material for Adolescents’ Emotion Regulation Strategies Questionnaire–Extended: Further Development and Associations With Mental Health Problems in Adolescence Click here for additional data file. Supplemental material, sj-docx-1-asm-10.1177_10731911231164619 for Adolescents’ Emotion Regulation Strategies Questionnaire–Extended: Further Development and Associations With Mental Health Problems in Adolescence by Gustaf Rådman, Benjamin Claréus and Daiva Daukantaitė in Assessment
