# Supplementary material for: Adolescents’ Emotion Regulation Strategies Questionnaire–Extended: Further Development and Associations With Mental Health Problems in Adolescence
Source: Assessment. 2023 Apr 13;31(2):482–501. doi: 10.1177/10731911231164619 (PMC10822064; doi:10.1177/10731911231164619)
Supplement: sj-docx-1-asm-10.1177_10731911231164619 – Supplemental material for Adolescents’ Emotion Regulation Strategies Questionnaire–Extended: Further Development and Associations With Mental Health Problems in Adolescence [file sj-docx-1-asm-10.1177_10731911231164619.docx]

**Supplementary material**

**Revising the scale**

Below we describe in more detail how we sought to improve on the original scale. Generally speaking, we looked at iterations of the original AERSQ and its larger item pool. Rather than focusing only on the items retained in the original final AERSQ, we took a broader focus attempting to further improve on the items from the broader pool of items. Details about the approach taken to each subscale is presented below. We do not describe the development of the new distraction subscale or the aggressive outlet subscale, since no counterpart is available in the original scale (the final distraction subscale is different enough from the old version to make comparisons futile).

*Rumination/negative thinking:*

The original items, although having good psychometric properties (α = .81, λ_min_ = .53; Zhou et al., 2020), we wished to improve on the specificity of the intended constructs, as well as improve the understandability for adolescents.

- “I think negative thoughts about myself” changed to “Think I am bad or worthless” explicitly stating what we intended with the phrase “negative thoughts” to avoid unnecessary confusion and multiple interpretations.
- Removed “I withdraw and keep to myself” because we wanted to emphasize processes that implied engagement with negative affect and not the other way around (even though withdrawal is most likely highly linked to rumination and negative thinking.
- Added “Think about things I’ve said or done (again and again)” to capture rumination more explicitly.
- Removed (1) “I think that I am badly treated by others” and (2) “I feel angry over having these feelings” to avoid items that, in our judgement, in part go outside the intended construct, involving processes such as (1) blaming of others, (2) feelings of aggression.

*Positive reorientation:*

For this scale, one of the main focuses was to avoid cross-loadings with the distraction subscale. We aimed to remove or change items that seemed to indicate avoidant or distractive patterns toward negative affect, and to focus on patterns of reappraisal.

- Changed “I try to find the positive aspects of what has happened” to “Try to find something positive in what has happened”, simplifying the language.
- Removed “I try to avoid thinking about my unpleasant feelings” because of its emphasis on avoidance rather than reorientation/reappraisal.
- Removed “I try to think about pleasant things and daydream” due to its indication of avoidance or distraction.
  - However, we chose to initially retain “I try to do something that will make me feel better” since it does not as explicitly imply distracting away from the felt emotions (depending on the activity).
- Added “Stay calm and think that it will pass.” aiming to cover adopting a positive frame of mind toward presence and future.
- Added “Move on and try to do things better next time” aiming to capture a positive but, in contrast to “Stay calm and think that it will pass.”, productive and active frame of mind.
- Added “Try to accept and be open about how I feel” aiming to capture open acceptance toward oneself and others (**this item was part of the original AERSQ during EFA**)**.**

*Creative expression (previously “Cultural activities”):*

With the rename of this subscale, we aimed to generalize the intended psychological process to that of creative endeavors presumably expressing one’s emotions. Consequently, we clustered several of the items from the previous version, as well as added new items.

- Removed “I read” to isolate the subscale to expressive activities.
- Replaced “I write a diary” with a more indirect form of literary expression: “Write texts (*e.g.,* stories, song lyrics, poems)” and a more general one: “Write down thoughts about how I feel” broadening the scope.
- Replaced “I draw, paint, play an instrument, or dance” with the two items “Draw or paint” and “Dance, play instruments or sing” separating activities related to visual art and musical art.

*Social support (previously “Communication”)*

The original scale only consisted of two items, both related to talking about felt emotions with a friend. By reconceptualizing this scale as “Social support”, we both broadened the construct slightly as well as narrowed down the intended ER function more concretely. Consequently, we also added new items to cover this broader construct better.

- Changed “I speak with friends on the phone” to “Call to talk to someone or chat with someone”, generalizing beyond those defined as friends and adding chatting.
- Changed “I speak with friends about how I feel” to “Tell someone else how I feel”, generalizing beyond those defined as friends.
- Added (1) “Seek support and comfort in others”, capturing consolation (2) “Ask others for advice or help” capturing advice seeking, (3) “Seek physical contact (*e.g.,* a hug)” capturing intimacy seeking.

| **Table S1**  *Comparison between AERSQ and AERSQ-E (italicized items were removed from final version).* | | | | | | | | |
| --- | --- | --- | --- | --- | --- | --- | --- | --- |
| **AERSQ** | | | | **AERSQ-E**  When things feel bad I… | | | | |
| **Rumination/negative thinking** | | | | **Rumination/negative thinking** | | | | |
|  | I think negative thoughts about myself | | |  | Think that I am bad or worthless | | | |
|  | I withdraw and keep to myself | | |  | Think about things I’ve said or done (again and again) | | | |
|  | I think that I am badly treated by others | | |  | Worry about what others might think of me | | | |
|  | I think that others are more fortunate than me | | |  | Think that others have it much better off than me | | | |
|  | I have the urge to physically hurt myself | | |  | *Want to hurt myself* | | | |
|  | I think that it is impossible to do anything about how I feel | | |  | *Think that it is impossible to do anything about how I feel.* | | | |
|  | I feel angry over having these feelings | | |  |  | | | |
| **Positive reorientation** | | | | **Positive reorientation** | | | | |
|  | I try to find the positive aspects of what has happened | | |  | Try to find something positive in what has happened | | | |
|  | I try to avoid thinking about my unpleasant feelings | | |  | Try in a calm manner to solve what made me feel bad | | | |
|  | I try to think about pleasant things and daydream | | |  | Stay calm and think that it will pass | | | |
|  | I try to do something that will make me feel better | | |  | *Find something to do that will make me feel better* | | | |
|  | - | | |  | Move on and try to do things better next time | | | |
|  | - | | |  | *Try to accept and be open about how I feel* | | | |
| **Cultural activities** | | | | **Creative expression** | | | | |
|  | I read | | |  | Create something that expresses how I feel | | | |
|  | I write a diary | | |  | Write texts (*e.g.*, stories, song lyrics, poems) | | | |
|  |  | | |  | Write down thoughts about how I feel | | | |
|  | I draw, paint, play an instrument, or dance | | |  | Draw or paint | | | |
|  | - | | |  | *Dance, play instruments or sing* | | | |
| **Communication** | | | | **Social support** | | | | |
|  | I speak with friends on the phone | | |  | *Call to talk to someone or chat with someone* | | | |
|  | I speak with friends about how I feel | | |  | Tell someone else how I feel | | | |
|  | - | | |  | Seek support and comfort in others | | | |
|  | - | | |  | Ask others for advice or help | | | |
|  | - | | |  | Seek physical contact (*e.g.*, a hug) | | | |
| **(Not included)** | | | | **Aggressive outlet** | | | | |
|  | - | | |  | Punch or kick on things | | | |
|  | - | | |  | Try to find something to break | | | |
|  | - | | |  | Argue or fight with people around me | | | |
|  | - | | |  | Want to hurt others (physically or mentally) | | | |
| **Distraction** | | **Distraction (initial revision)** | | | | **Distraction (final version)** | | |
|  | I write to or chat online with others |  | Hang out on social media (e.g., Instagram, TikTok, Snapchat) | | |  | Try to think about something else |  |
|  | I play video games or computer games |  | Play games (e.g., videogames or computer games) | | |  | Distract myself with something to do |  |
|  | I listen to music or watch TV or online videos |  | Watch something (e.g., movies, tv-shows, videos, streaming) | | |  | Try to forget that which makes me feel bad |  |
|  | I sleep, rest and relax |  | Listen to music | | |  | *Pretend like the things I feel do not exist.* |  |
|  | I eat something |  | Cook, bake and/or eat something | | |  | *Avoid things that remind me of my feelings.* |  |
|  |  |  | Do something where I move my body (*e.g.*, walk, bicycle, run) | | |  |  |  |

**Table S2**

*Exploratory Factor Analysis on the initial 33 item version of AERSQ-E.*

|  | Sample 1-2  Factor | | | | | |  |
| --- | --- | --- | --- | --- | --- | --- | --- |
| Item (in English translation) | 1 | 2 | 3 | 4 | 5 | 6 | |
| R1. Think about things I have said or done (again and again) | **.74** | .04 | .04 | -.04 | -.01 | .11 | |
| R2. Think that I am bad or worthless | **.72** | -.15 | .06 | .08 | -.03 | -.09 | |
| R3. Worry about what others might think of me | **.70** | -.03 | .04 | .00 | .02 | .05 | |
| R4. Believe that others have it much better off than me | **.54** | .06 | -.01 | .16 | -.06 | .04 | |
| P1. Try to find something positive in what has happened | -.04 | **.67** | .01 | .03 | .03 | -.02 | |
| P2. Move on and try to do things better next time | .02 | **.64** | -.11 | -.11 | .01 | .05 | |
| P3. Try in a calm manner to solve what made me feel bad | -.12 | **.61** | -.01 | -.04 | .00 | -.08 | |
| P4. Stay calm and think that it will pass | -.02 | **.62** | .03 | -.09 | .13 | .01 | |
| C1. Create something that expresses how I feel | .02 | .01 | **.66** | .07 | .01 | -.03 | |
| C2. Write texts (*e.g.*, stories, song lyrics, poems) | -.02 | -.09 | **.60** | .00 | .02 | -.01 | |
| C3. Draw or paint | .11 | .09 | **.54** | -.04 | -.06 | .16 | |
| C4. Write down thoughts about how I feel | .13 | -.20 | **.49** | -.04 | .22 | .01 | |
| C5. Dance, play instruments or sing | .03 | .22 | **.40** | .10 | .00 | .06 | |
| A1. Punch or kick on things | -.14 | .00 | .06 | **.66** | -.11 | -.07 | |
| A2. Try to find something to break | -.04 | -.09 | .10 | **.62** | -.11 | -.10 | |
| A3. Argue or fight with people around me | .18 | .02 | -.04 | **.64** | .12 | .09 | |
| A4. Want to hurt others (physically or mentally) | .09 | -.05 | .02 | **.57** | .01 | .04 | |
| A5. Whine, mope or get unpleasant toward other people | .19 | -.07 | -.11 | **.51** | .14 | .23 | |
| S1. Tell someone else how I feel | -.12 | -.06 | -.01 | -.01 | **.89** | .06 | |
| S2. Seek support and comfort in others | .14 | .03 | -.02 | -.05 | **.84** | -.05 | |
| S3. Ask others for advice or help | -.02 | .23 | .04 | .09 | **.64** | -.07 | |
| S4. Seek physical contact (*e.g.*, a hug) | .08 | .13 | .08 | .04 | **.60** | -.06 | |
| S5. Call and talk to someone or chat with someone | -.14 | -.09 | .12 | -.01 | **.53** | .30 | |
| P5. Try to accept and be open with how I feel | -.10 | .28 | .10 | -.05 | **.42** | -.07 | |
| D6. Watch something (*e.g.*, a movie, television shows, streaming) | .07 | .12 | .15 | -.06 | -.04 | **.59** | |
| D7. Hang out on social media (*e.g.*, Instagram, TikTok, Snapchat) | .12 | -.13 | -.09 | .13 | .04 | **.56** | |
| D8. Play games (*e.g.*, video or computer games) | -.22 | .13 | -.03 | .05 | -.29 | .24 | |
| D9. Listen to music | .12 | -.01 | .26 | .06 | .02 | .34 | |
| D1. Cook food, bake and/or eat something | .14 | .12 | .17 | .09 | -.02 | .28 | |
| D11. Do something where I move my body (*e.g.*, walk, bicycle, run) | -.04 | .39 | .05 | .05 | .12 | .21 | |
| R5. Want to hurt myself | .11 | -.32 | .21 | .17 | -.01 | -.12 | |
| R6. Believe there is nothing to be done about how I feel | .36 | -.21 | .07 | .18 | .11 | .03 | |
| P6. Find something to do that makes me feel better | -.19 | .31 | .10 | .01 | .06 | .28 | |

*Note:* Items that were excluded from the measure before data collection in Sample 3 had commonalities between 1.8 and 3.4.

| **Table S3**.  *Confirmatory Factor Analysis summary of fit statistics including Latent Factor Loadings, Modifications and Fit Indices.* | | | | |
| --- | --- | --- | --- | --- |
|  | | **Sample** | | |
| **Factor/question** | | 4 + 5 | 6 | Combined |
| Rumination/negative thinking | |  |  |  |
| R1. Think about things I have said or done (again and again) | .75 | .63 | .67 |  |
| R2. Think that I am bad or worthless | .83 | .77 | .79 |  |
| R3. Worry about what others might think of me | .74 | .75 | .75 |  |
| R4. Believe that others have it much better off than me | .53 | .59 | .56 |  |
| Positive reorientation | |  |  |  |
| P1. Try to find something positive in what has happened | .68 | .67 | .69 |  |
| P2. Move on and try to do things better next time | .71 | .72 | .71 |  |
| P3. Try in a calm manner to solve what made me feel bad | .66 | .68 | .68 |  |
| P4. Stay calm and think that it will pass | .69 | .69 | .69 |  |
| Creative expression | |  |  |  |
| C1. Create something that expresses how I feel | .52 | .64 | .57 |  |
| C2. Write texts (*e.g.*, stories, song lyrics, poems) | .67 | .89 | .84 |  |
| C3. Draw or paint | .53 | .41 | .40 |  |
| C4. Write down thoughts about how I feel | .64 | .80 | .77 |  |
| Aggressive outlet | |  |  |  |
| A1. Punch or kick on things | .51 | .31 | .41 |  |
| A2. Try to find something to break | .53 | .39 | .46 |  |
| A3. Argue or fight with people around me | .81 | .87 | .82 |  |
| A4. Want to hurt others (physically or mentally) | .71 | .54 | .62 |  |
| Social support | |  |  |  |
| S1. Tell someone else how I feel | .81 | .81 | .81 |  |
| S2. Seek support and comfort in others | .80 | .88 | .85 |  |
| S3. Ask others for advice or help | .67 | .73 | .71 |  |
| S4. Seek physical contact (*e.g.*, a hug) | .52 | .67 | .62 |  |
| Distraction | |  |  |  |
| D1. Try to think about something else | .41 | .88 | .78 |  |
| D2. Distract myself with something to do | .73 | .54 | .57 |  |
| D3. Try to forget that which makes me feel bad | .54 | .47 | .46 |  |
| Latent factor covariances | |  |  |  |
| Rumination/negative thinking ~ Positive reorientation | -.33 | -.43 | -.37 |  |
| Rumination/negative thinking ~ Creative expression | .40 | .35 | .35 |  |
| Rumination/negative thinking ~ Aggressive outlet | .47 | .44 | .46 |  |
| Rumination/negative thinking ~ Social support | -.02 | .30 | .17 |  |
| Rumination/negative thinking ~ Distraction | .00 | .03 | .03 |  |
| Positive reorientation ~ Creative expression | .04 | -.01 | .03 |  |
| Positive reorientation ~ Aggressive outlet | -.46 | -.43 | -.44 |  |
| Positive reorientation ~ Social support | .44 | .21 | .33 |  |
| Positive reorientation ~ Distraction | .61 | .48 | .56 |  |
| Creative expression ~ Aggressive outlet | .19 | .18 | .19 |  |
| Creative expression ~ Social support | .19 | .31 | .28 |  |
| Creative expression ~ Distraction | .13 | .01 | .07 |  |
| Aggressive outlet ~ Social support | -.02 | .09 | .03 |  |
| Aggressive outlet ~ Distraction | -.17 | -.16 | -.17 |  |
| Social support ~ Distraction | .22 | .27 | .27 |  |
| Modifications | |  |  |  |
| A1. (“Punch or kick on things”)   ~ A2. (“Try to find something to break”) | .60 | .63 | .62 |  |
| Model fit* | |  |  |  |
| *N* | 252 | 340 | 592 |  |
| RMSEA | .046 | .057 | .053 |  |
| RMSEA 95% CI | .035, .055 | .050, .064 | .048, .058 |  |
| SRMR | .063 | .064 | .058 |  |
| χ^2^ (*df* = 214) | 326.588 | 45.338 | 57.101 |  |
| CFI | .935 | .913 | .920 |  |
| *Based on the modified model described under Results. | | | | |
